# On the stability of the exact solutions of the dual-phase lagging model of heat conduction

**DOI:** 10.1186/1556-276X-6-327

**Published:** 2011-04-13

**Authors:** Jose Ordonez-Miranda, Juan Jose Alvarado-Gil

**Affiliations:** 1 Departamento de Física Aplicada, Centro de Investigación y de Estudios Avanzados del I.P.N.-Unidad Mérida. Carretera Antigua a Progreso km. 6, A.P. 73 Cordemex, C.P. 97310, Mérida, Yucatán, México

## Abstract

The dual-phase lagging (DPL) model has been considered as one of the most promising theoretical approaches to generalize the classical Fourier law for heat conduction involving short time and space scales. Its applicability, potential, equivalences, and possible drawbacks have been discussed in the current literature. In this study, the implications of solving the exact DPL model of heat conduction in a three-dimensional bounded domain solution are explored. Based on the principle of causality, it is shown that the temperature gradient must be always the cause and the heat flux must be the effect in the process of heat transfer under the dual-phase model. This fact establishes explicitly that the single- and DPL models with different physical origins are mathematically equivalent. In addition, taking into account the properties of the Lambert W function and by requiring that the temperature remains stable, in such a way that it does not go to infinity when the time increases, it is shown that the DPL model in its exact form cannot provide a general description of the heat conduction phenomena.

## Introduction

Nanoscale heat transfer involves a highly complex process, as has been witnessed in the last years in which remarkable novel phenomena related to very short time and spatial scales, such as enhancement of thermal conductivity in nanofluids, granular materials, thin layers, and composite systems among others, have been reported [1-5]. The traditional approach to deal with these phenomena has been to use the Fourier heat transfer equation. This methodology has proven to be extensively useful in the analysis of heat transport in a great variety of physical systems, however, when applied to highly heterogeneous systems or when the time and space scale are very short, they show serious inconsistencies [6,7]. In order to understand the nanoscale heat transfer, a great diversity of novel theoretical approaches have been developed [3,5,7,8]. In particular, when analyzing two-phase systems, one of the simplest heat conduction models that considers the microstructure is known as the two-equation model [9,10], which has been developed writing the Fourier law of heat conduction [11] for each phase and performing a volume averaging procedure [9]. This model takes into account the porosity of the component phases as well as their interface effects by means of two coefficients [12]. Besides, it has been shown that the two-equation model is equivalent to the one-equation model known as the dual-phase lagging (DPL) model, in which the microstructural effects are taken into account by means of two time delays [3,10,13-15]. DPL model have been proposed to surmount the well-known drawbacks of the Fourier law and the Cattaneo equation of heat conduction [7], and establishes that either the temperature gradient may precede the heat flux or the heat flux may precede the temperature gradient. Mathematically, this is written in the form(1)

where  is the position vector, *t *is the time,  is the heat flux vector, *T*[K] is the absolute temperature, *k*[W.m^-1^.K^-1^] is the thermal conductivity, Ʈ_*q *_is the phase lag of the heat flux, and Ʈ_*T *_is the phase lag of the temperature gradient. For the case of Ʈ_*q*_>Ʈ_*T*_, the heat flux (effect) established across the material is a result of the temperature gradient (cause); while for Ʈ_*q*_<Ʈ_*T*_, the heat flux (cause) induces the temperature gradient (effect). Notice that when Ʈ_*q *_= Ʈ_*T*_, the response between the temperature gradient and the heat flux is instantaneous and Equation 1 reduces to Fourier law except for a trivial shift in the time scale. In addition, note that for Ʈ_*T *_= 0; the DPL model reduces to the single-phase lagging (SPL) model [3]. The time delay Ʈ_*q *_is interpreted as the relaxation time due to the fast-transient effects of thermal inertia, while the phase lag Ʈ_*T *_represents the time required for the thermal activation in micro-scale [3]. For the case of composite materials, the phase lag Ʈ_*q *_takes into account the time delay due to contact thermal resistance among the particles, while Ʈ_*T *_is interpreted as the time required to establish the temperature gradient through the particles [12,16]. The lagging behavior in the transient process is caused by the finite time required for the microscopic interactions to take place. This time of response has been claimed to be in the range of a few nanoseconds in metals and up to the order of several seconds in granular matter [3]. In this last case, due to the low-conducting pores among the grains and their interface thermal resistance.

The thermal conductivity is an intrinsic property of each material which measures its ability for the transfer of heat and is determined by the kinetic properties of the energy carriers and the material microstructure [6,17]. Under the framework of Boltzmann kinetic theory [3,6], it can be shown that the thermal conductivity is directly proportional to the group velocity and mean free path of the energy carriers (electrons and phonons). These parameters depend strongly on the material temperature, due to the multiple scattering processes involved among energy carriers and defects, such as impurities, dislocations, and grain boundaries, [6,18]. Thus, in general; thermal conductivity exhibits complicated temperature dependence. However, in many cases of practical interest, the thermal conductivity can be considered independent of the temperature for a considerable range of operating temperatures [3,6,11]. Based on this fact and to keep our mathematical approach tractable, we assume that thermal conductivity is a temperature-independent parameter.

Phase lags represent the time parameters required by the material to start up the heat flux and temperature gradient, after a thermal excitation has been imposed; larger phase lags are expected in material with smaller thermal conductivities, as is the case of granular matter [3]. Materials, in which the temperature gradient phase lag dominates, show a strong attenuation of the neat heat flux. In this case, the behavior is dominated by parabolic terms of the heat transport equation. In contrast, materials in which the heat flux phase lag is dominant show a slight attenuation of the heat flux, implying that a hyperbolic Cattaneo-Vernotte heat propagation is present. For a further discussion of the relationship between thermal conductivity and phase lags, Tzou's book [3] is recommended.

It is convenient to take into account that the heat flux and temperature gradient shown in Equation 1 are the local responses within the medium. They must not be confused with the global quantities specified in the boundary conditions. When a heat flux (as a laser source) is applied to the boundary of a solid medium, the temperature gradient established within the medium can still precede the heat flux. The application of the heat flux at the boundary does not guarantee the precedence of the heat flux vector to the temperature gradient at all. In fact, whether the heat flux vector precedes the temperature gradient or not depends on the combined effects of the thermal loading and thermal properties of the materials, as was explained by Tzou [3]. In this way, the DPL model should provide a comprehensive treatment of the heterogeneous nature of composite media [3,13].

It has been shown that under the DPL model and in absence of internal heat sources, the temperature satisfies the following differential-difference equation [19-22]:(2)

where *α*[m^2^.s^-1^] is the thermal diffusivity of the medium, and *Ʈ *= *Ʈ_q_-Ʈ_T _*is the difference of the phase lags. Equation 2 shows explicitly that the DPL and SPL models, both in their exact form, are entirely equivalent, when *Ʈ*> 0(*Ʈ_q_-Ʈ_T_*)[19].

The solutions of Equation 2 for some geometries have been explored [19-22]. In the time domain, Jordan et al. [19] and Quintanilla and Jordan [22] have shown that the SPL model, in its exact form, can lead to instabilities with respect to specific initial values. Additionally, in the frequency domain, using a modulated heat source, Ordonez-Miranda and Alvarado-Gil [21] have shown that the if the DPL model is valid, its applicability must be restricted to frequency-interval strips, which are determined only by the difference of the time delays *Ʈ *= *Ʈ_q_-Ʈ_T_*. These studies have pointed out that the usefulness of the Cattaneo-Vernotte and DPL exact models is limited.

In this study, by means of the method of separation of variables, the solution of Equation 2 is obtained in a bounded domain. It is shown that, for any kind of homogeneous boundary conditions, its solutions go to infinity in the long time domain. This explosive characteristic of the temperature predicted by Equation 2 indicates that the DPL model, in its exact form, cannot be considered as a valid model of heat conduction.

## Mathematical formulation and solutions

The general solution of Equation 2 in a three-dimensional closed region of finite volume *V *and boundary surface ∂*V *is going to be obtained in this section. The initial-boundary value problem to be solved can be written as follows:(3a)(3b)(3c)

where *a *and *b *are two constants and  is a unit normal vector pointing outward of the boundary surface ∂*V*. Note that the boundary conditions in Equation 3a imply the specification of the temperature and heat flux at ∂*V *and they reduce to the Dirichlet (Neumann) problem for *b *= 0 (*a *= 0) [5]. On the other hand, the initial condition is specified in the pre-interval [-Ʈ,0] to define the time derivative of the temperature in the interval [0,Ʈ]. This is a common characteristic of the delay differential equations, as Equation 3a [23]. In many common situations the initial history function  may be considered as a constant.

According to the method of separation of variables, a solution of the form(4)

is proposed. After inserting Equation 4 into Equations 3a, b, it is obtained that(5a)(5b)(5c)

where the integer subscript *m *= 1,2,3,... has been inserted in view that Equations 5a, b defined an eigenvalue (Sturm-Liouville) problem [5], and *λ_m _*is the eigenvalue associated with the eigenfunction *ψ_m_*. As an example, in the case of one-dimensional heat conduction across a finite region 0 ≤*x*≤*l*, nine possible combinations of the boundary conditions given by Equation 5b can be found [5]. One of these combinations occurs when both surfaces *x *= 0 and *x *= *l *are insulated (). After applying these particular boundary conditions to the solution of Equation 5a, it is found that its eigenvalues are determined by . Similar results can be obtained for the other combinations of boundary conditions as well as for more complex geometries [5]. In general, all the eigenvalues are real and positive, and they go to infinity when *m*→∞[5]. In this way, by the principle of superposition, the general solution of Equation 3a-c can be written as(6)

where Equation 5c can be solved assuming that *P_m_*(*t*) = e*x*p(*st*) is its solution for some value of *s*. This provides the relationship(7)

whose solutions can be expressed in a closed form by means of the Lambert W function as follows [24]:(8)

where *r *= 0,± 1,± 2,... indicates a specific branch of the complex-valued function *W_r_*. For *y*≠-*e*^-1^, all the branches of *W_r_(y) *are different; while for *y *= -*e*^-1^, the branches *W*_-1_(*y*) = *W*_0_(*y*) = -1 and the others have different values among them. In this way, the general solution of Equation 5c is given by(9a)(9b)

where *αƮλ_M _*= *e*^-1 ^and the constants *C_m,r _*and *D_m,r _*can be determined by expanding Equation 3c in terms of the orthogonal set of eigenfunctions {*ψ_m_*} as follows:(10)

In this way, for -Ʈ≤*t*≤ 0(11)

is satisfied. However, in practice the determination of the coefficients *C_m,r _*and *D_m,r _*by means of Equation 11 may be complicated. This can be avoided by solving Equation 5c using the Laplace transform method. After taking the Laplace transform of Equation 5c, and using Equation 11, it is obtained that in the Laplace domain, the function *P_m_(s)*≡*L[P_m_(t)*] is given by(12)

where *B_m_(s)*≡*L[b_m_(t)*] for the time domain -τ≤*t*≤ 0. Using the complex inversion formula of the Laplace transform [5], it is obtained that(13)

where *R*[] stands for the residue of its argument. Given that the poles of Equation 12 are determined by equating to zero its denominator, these poles *s_r,m _*are determined by Equation 8. Note that all the poles are simple if *αƮλ_m_*≠*e*^-1^, and there is a double pole for *αƮλ_m _*= *e*^-1^, at *r *= -1,0. In this way, after calculating the residues involved in Equation 13 and comparing Equations 9a, b with Equation 13 it is found that(14a)(14b)(14c)(14d)

where the parameters *s_r,m _*are given by Equation 8 and the prime (') on *B_m _*indicates derivative with respect to its argument. For the particular case in which the initial history function does not depend on time, the coefficient *b_m _*= constant ≡*b*_0 _and Equations 14a-d reduce to(15a)(15b)(15c)(15d)

which agree with the previous results of Jordan et al. [19]. It is interesting to note that by requiring that *P_m_*(0) = *b*_0 _in Equation 9a, the following property of the Lambert *W *function is obtained(16)

where *y* ≡ -*αƮλ_m_*. Using appropriate software, Equation 16 can be verified to be valid not only for the roots of Equation 7, but also for any value of *y*.

## Analysis of the results

**Figure 1:**
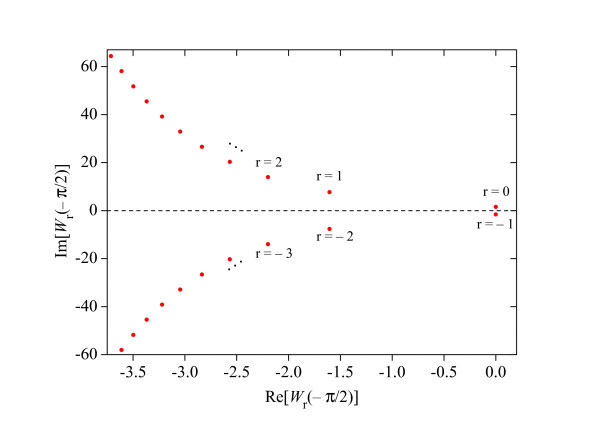
**Distribution of the imaginary values of *W_r_*(*y*) with respect to its real values, at *y *= π/2**.

In this section, the time-dependent part of the temperature is going to be analyzed in two key points, as follows:

• According to Equation 5c, the temporal rate of change of *P_m_(t*) (and therefore of the temperature) is determined by its value at the past (future), if Ʈ> 0 (Ʈ< 0). Based on the principle of causality, the future cannot determine the past, and therefore the DPL model in its exact form (Equation 1) must take into account the constraint *Ʈ *= *Ʈ_q_-Ʈ_T_*> 0. In this way, the DPL and SPL models are fully equivalent between them [3,5]. This fact is in strong contrast to the values of the phase lags, reported by Tzou [3]. By expanding both sides of Equation 1 in a Taylor series and considering a first-order approximation in the phase lags, this author found that *Ʈ_T _*= 100 *Ʈ_q _*for metals. This discrepancy with the causality principle indicates that the predictions of the DPL model in its approximate and exact forms may be remarkably different. This fact reveals that the small-phase lags can have great effects, as it has been shown in the theory of delayed differential equations [23].

• Based on Equation 9a and taking into account that the principle of causality demands that *Ʈ*> 0, as has been discussed in above, it can be observed that the temperature remains stable (finite) for large values of time, if the following condition is satisfied(17)

where *Re*[] stands for the real part of its argument. For *y *= π/2, Figure 1 shows that the larger real parts of *W_r_(y*) are given when *r *= -1,0. In general, after a graphical analysis of the Lambert *W *function, it can be concluded that [24]. Based on this result, Equation 17 can be replaced by(18)

Given that ,  and  (see Figure 1), the inequality (18) is satisfied if and only if(19)

which represent the stability condition of the temperature for long times.

Taking into account that *λ_m_*→∞ for *m*→∞, it can be observed that the condition (19) cannot be satisfied for arbitrarily large values of *m*. The only way to solve this would be by imposing that *m*<*m_max_*, in such a way that  however, under this restriction on the values of *m*, the initial condition could not be satisfied (Equation 10). In this way, it is concluded that the DPL model in its exact form establishes that the temperature increases without limit when the time grows, which is physically unacceptable. This divergent behavior of the temperature, in the DPL model at long times, is the direct consequence of having introduced the phase lags. Even though the effects of these parameters are obviously very important for short time scales, according to our results (see Equations 1 and 9a, b), the assumption of taking them as different from zero implies non-physical behavior at large time scales. Therefore, the DPL model, in its exact form, cannot be a valid formalism for heat conduction analysis in the complete time scale. It is expected that the correct model of heat conduction at both short and large scales could be derived from the Boltzmann transport equation under the relaxation time approximation [6].

## Conclusions

By combining the methods of separation of variables and the Laplace transform, the exact solution of the DPL model of heat conduction in a three-dimensional bounded domain has been obtained and analyzed. According to the principle of causality, it has been shown that the temperature gradient must precede the heat flux. In addition, based on the properties of the Lambert *W *function, it has been shown that the DPL model predicts that the temperature increases without limit when the time goes to infinity. This unrealistic prediction indicates that the DPL model, in its exact form, does not provide a general description of the heat conduction phenomena for all time scales as had been previously proposed.

## Abbreviations

DPL: dual-phase lagging; SPL: single-phase lagging.

## Competing interests

The authors declare that they have no competing interests.

## Authors' contributions

JOM carried out the mathematical calculations, participated in the interpretations of the results and drafted the manuscript. JJAG conceived of the study, participated in the analysis of the results and improved the writing of the manuscript. All authors read and approved the final manuscript.
